# Long Noncoding RNA and Predictive Model To Improve Diagnosis of Clinically Diagnosed Pulmonary Tuberculosis

**DOI:** 10.1128/JCM.01973-19

**Published:** 2020-06-24

**Authors:** Xuejiao Hu, Shun Liao, Hao Bai, Shubham Gupta, Yi Zhou, Juan Zhou, Lin Jiao, Lijuan Wu, Minjin Wang, Xuerong Chen, Yanhong Zhou, Xiaojun Lu, Tony Y. Hu, Zhaolei Zhang, Binwu Ying

**Affiliations:** a Department of Laboratory Medicine, West China Hospital, Sichuan University, Chengdu, People’s Republic of China; b Division of Laboratory Medicine, Guangdong Provincial People’s Hospital, Guangdong Academy of Medical Sciences, Guangzhou, People’s Republic of China; c Department of Computer Science, University of Toronto, Toronto, Ontario, Canada; d Department of Molecular Genetics, University of Toronto, Toronto, Ontario, Canada; e The Donnelly Centre for Cellular and Biomolecular Research, University of Toronto, Toronto, Ontario, Canada; f Department of Respiratory and Critical Care Medicine, West China Hospital, Sichuan University, Chengdu, People’s Republic of China; g Center for Cellular and Molecular Diagnostics, Department of Biochemistry and Molecular Biology, School of Medicine, Tulane University, New Orleans, Louisiana, USA; Carter BloodCare and Baylor University Medical Center

**Keywords:** clinically diagnosed pulmonary tuberculosis, electronic health record, lncRNA, nomogram

## Abstract

Clinically diagnosed pulmonary tuberculosis (PTB) patients lack microbiological evidence of Mycobacterium tuberculosis, and misdiagnosis or delayed diagnosis often occurs as a consequence. We investigated the potential of long noncoding RNAs (lncRNAs) and corresponding predictive models to diagnose these patients. We enrolled 1,764 subjects, including clinically diagnosed PTB patients, microbiologically confirmed PTB cases, non-TB disease controls, and healthy controls, in three cohorts (screening, selection, and validation). Candidate lncRNAs differentially expressed in blood samples of the PTB and healthy control groups were identified by microarray and reverse transcription-quantitative PCR (qRT-PCR) in the screening cohort. Logistic regression models were developed using lncRNAs and/or electronic health records (EHRs) from clinically diagnosed PTB patients and non-TB disease controls in the selection cohort. These models were evaluated by area under the concentration-time curve (AUC) and decision curve analyses, and the optimal model was presented as a Web-based nomogram, which was evaluated in the validation cohort. Three differentially expressed lncRNAs (*ENST00000497872*, *n333737*, and *n335265*) were identified. The optimal model (i.e., nomogram) incorporated these three lncRNAs and six EHRs (age, hemoglobin, weight loss, low-grade fever, calcification detected by computed tomography [CT calcification], and interferon gamma release assay for tuberculosis [TB-IGRA]). The nomogram showed an AUC of 0.89, a sensitivity of 0.86, and a specificity of 0.82 in differentiating clinically diagnosed PTB cases from non-TB disease controls of the validation cohort, which demonstrated better discrimination and clinical net benefit than the EHR model. The nomogram also had a discriminative power (AUC, 0.90; sensitivity, 0.85; specificity, 0.81) in identifying microbiologically confirmed PTB patients. lncRNAs and the user-friendly nomogram could facilitate the early identification of PTB cases among suspected patients with negative M. tuberculosis microbiological evidence.

## Abstract (precis)

Clinically diagnosed pulmonary tuberculosis (PTB) patients lack microbiological evidence of Mycobacterium tuberculosis, and misdiagnosis or delayed diagnosis often occurs as a consequence. We investigated the potential of long noncoding RNAs (lncRNAs) and corresponding predictive models to diagnose these patients. We enrolled 1,764 subjects, including clinically diagnosed PTB patients, microbiologically confirmed PTB cases, non-TB disease controls, and healthy controls, in three cohorts (screening, selection, and validation).

## INTRODUCTION

Tuberculosis (TB) is the leading cause of death from an infectious agent (1), but only 56% of pulmonary tuberculosis (PTB) cases reported to the WHO in 2017 were bacteriologically confirmed. Thus, approximately one-half of all PTB cases are clinically diagnosed worldwide, and this proportion can reach 68% in China (1). Clinically diagnosed PTB cases are symptomatic but lack evidence of Mycobacterium tuberculosis infection by smear microscopy, culture, or nucleic acid amplification tests (1–3). The diagnostic procedure for clinically diagnosed PTB is inadequate and time-consuming and often results in misdiagnosis or delayed diagnosis (3), leading to an increased risk of morbidity and mortality (4) or overtreatment (5). There is thus an urgent need to develop rapid and accurate strategies to diagnose PTB cases without microbiological evidence of M. tuberculosis (6, 7). The exploration of effective host immune response signatures represents an attractive approach for this type of assay.

Long noncoding RNAs (lncRNAs) can function as critical regulators of inflammatory responses to infection, especially for T-cell responses (8, 9). Increasing evidence indicates that blood lncRNA expression profiles are closely associated with TB disease (10–12), suggesting that lncRNAs could function as potential noninvasive biomarkers for TB detection. However, previous studies have suffered from small sample sizes (ranging from 66 to 510) and a lack of independent validation.

Recent effort has focused on establishing clinical prediction rules or predictive models for TB diagnosis based on patients’ electronic health record (EHR) information (13–16). Such models can cost-effectively facilitate PTB diagnosis with a limited number of clinical-radiological predictors. For example, a 6-signature model described previously by Griesel et al. (a cough lasting ≥14 days, the inability to walk unaided, a temperature of >39°C, chest radiograph assessment, hemoglobin level, and white cell count) attained an area under the concentration-time curve (AUC) of 0.81 (95% confidence interval [CI], 0.80 to 0.82) in seriously ill HIV-infected PTB patients (13). However, despite these advances, current EHR models remain insufficient for precise TB diagnosis. Compelling studies have proposed that models incorporating biomarkers and EHR information attain better performance for the prediction of sepsis (17) and abdominal aortic aneurysm (18). We previously reported that combining exosomal microRNAs and EHRs in the diagnosis of tuberculous meningitis (TBM) achieved AUCs of up to 0.97, versus an AUC of 0.67 obtained using EHRs alone (19). Based on these studies, we hypothesized that combining lncRNAs with well-defined EHR predictors could be used to develop improved predictive models to identify PTB cases that lack microbiological evidence of M. tuberculosis infection.

This study was therefore performed to investigate the diagnostic potential of lncRNAs and predictive models incorporating lncRNA and EHR data for the identification of PTB cases without microbiological evidence of M. tuberculosis. This study also explored the diagnostic potential of lncRNA candidates and the optimal model for microbiologically confirmed PTB.

## MATERIALS AND METHODS

### Study design.

**FIG 1:**
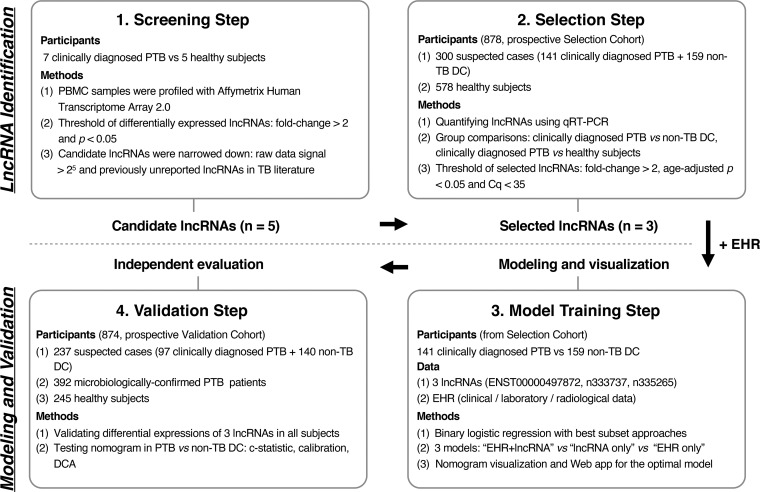
Overview of the strategy for investigating lncRNAs and prediction models for clinically diagnosed PTB patients. Abbreviations: PTB, pulmonary tuberculosis; PBMC, peripheral blood mononuclear cell; non-TB DC, nontuberculosis disease control; DE, differentially expressed; EHR, electronic health record; DCA, decision curve analysis.

We performed this study through a four-stage approach. lncRNAs that were differentially expressed (DE) between clinically diagnosed PTB patients and healthy subjects were profiled by microarray in the screening step. The expression levels of the top five lncRNAs were then analyzed in a large prospective cohort in the selection step of the study, which reduced the number of lncRNAs from 5 to 3 based on expression differences among groups. In the model training step, lncRNAs and EHRs were used to develop predictive models for clinically diagnosed PTB patients and nontuberculosis disease control (non-TB DC) patients, and the optimal model was visualized as a nomogram. Finally, we validated lncRNAs and the nomogram in a prospective cohort, including both clinically diagnosed PTB and microbiologically confirmed PTB cases. The study strategy is shown in Fig. 1.

### Subject enrollment.

**(i) Screening cohort.** We retrospectively collected 7 age- and gender-matched PTB cases and 5 healthy controls as the screening cohort. They were 6 males and 6 females aged 22 to 59 years. PTB cases were clinically confirmed PTB patients with positive TB symptoms, negative microbiological evidence of TB, and a good response to anti-TB therapy. Healthy subjects had a normal physical examination and no history of TB.

**(ii) Selection and validation cohorts.** Inpatients with clinical-radiological suspicion of PTB but lacking microbiological evidence of TB were prospectively enrolled from West China Hospital between December 2014 and May 2017. The inclusion criteria for highly suspected patients were new patients with (i) high clinical-radiological suspicion of PTB, (ii) anti-TB therapy for <7 days on admission, (iii) negative microbiological evidence of TB (i.e., at least two consecutive negative smears, one negative M. tuberculosis DNA PCR result, and one negative culture result), (iv) an age of ≥15 years, and (v) no severe immunosuppressive disease, HIV infection, or cardiac or renal failure. Two experienced pulmonologists reviewed and diagnosed all presumptive PTB patients. According to the Chinese diagnostic criteria for PTB, final diagnoses for all cases were based on the combination of clinical assessment, radiological and laboratory results, and response to treatment (1, 2) (see Appendix S1 in the supplemental material). A 12-month follow-up through telephone or WeChat was used to confirm the classification of clinically diagnosed PTB patients and non-TB patients. Detailed descriptions of patients’ symptoms and recruitment, inclusion and exclusion criteria, laboratory examinations, diagnostic criteria and procedures, treatment, and sample size estimates are provided in Appendices S1 and S2 in the supplemental material. We also enrolled microbiologically confirmed PTB cases in the validation cohort. Healthy subjects were simultaneously recruited from a pool of healthy individuals with a normal physical examination and no history of TB.

The selection cohort was comprised of 878 participants (141 clinically diagnosed PTB cases, 159 non-TB DCs, and 578 healthy subjects), and the validation cohort had 874 participants (97 clinically diagnosed PTB cases, 392 microbiologically confirmed PTB cases, 140 non-TB DCs, and 245 healthy subjects). Details of the non-TB DCs are listed in Table S1 in the supplemental material. Ethics approval was obtained from the Clinical Trials and Biomedical Ethics Committee of West China [approval no. 2014(198)]. Informed consent was obtained from every participant.

### lncRNA detection.

**(i) RNA isolation and cDNA preparation.** Peripheral blood mononuclear cell (PBMC) samples were isolated from 3-ml fresh blood samples from each participant using a human lymphocyte separation tube kit (Dakewe Biotech Company Limited, China). Total RNA was extracted from PBMC isolates using TRIzol reagent (Invitrogen, USA). RNA concentration and purity were evaluated spectrophotometrically, and RNA integrity was determined using agarose gel electrophoresis (Fig. S1A). The PrimeScript RT reagent kit with gDNA Eraser (TaKaRa, Japan) was used to remove contaminating genomic DNA and synthesize cDNA. All the kits were used according to the manufacturers’ instructions.

**(ii) lncRNA microarray profiling.** lncRNA profiles were detected using Affymetrix human transcriptome array 2.0 chips based on a standard protocol (20). Raw data were normalized using the robust multiarray average expression measure algorithm. DE lncRNAs with *P* values of <0.05 and fold changes of >2 were identified using empirical Bayes-moderated *t* statistics and presented by hierarchical clustering and a volcano plot (21).

**(iii) qRT-PCR for lncRNAs.** Three lncRNAs were amplified using the following primers: 5′-TTCCTCACCCTCTTCCTGCT-3′ (forward) and 5′-AAGGCATGTGAGTAAGGGCG-3′ (reverse) for *ENST00000497872*, 5′-GCAGAAAGCAAGGACCAA-3′ (forward) and 5′-GGATGAGCAGCGATGAAG-3′ (reverse) for *n333737*, and 5′-CGCAGAAGTAAGTAGCCGGG-3′ (forward) and 5′-ACTGGATGAGCGTGAAGTGG-3′ (reverse) for *n335265* (Table S2). A final 10-μl-volume reaction mixture for reverse transcription-quantitative PCR (qRT-PCR) included 5 μl of SYBR Premix Ex *Taq* II (TaKaRa, China), 0.5 μl of 10 μM forward and reverse primers, 3 μl of double-distilled water (ddH_2_O) (PCR grade), and 1 μl of template cDNA. The cycling program consisted of 95°C for 1 min, followed by 35 cycles at 95°C for 10 s, 56 to 62ºC for 30 s, and 72°C for 60 s. The samples were denatured at 95°C for 30 s and then heated to 65°C for 30 s at a rate of 0.2°C/s. The ddH_2_O negative control and blank control in each reaction showed no detectable signals, ensuring the lack of contamination or nonspecific products. lncRNA expression was measured in a blind fashion, normalized to the endogenous control glyceraldehyde-3-phosphate dehydrogenase (GAPDH) gene, and calculated according to the $2^{-\Delta \Delta C_{q}}$ method, where a quantification cycle (*C_q_*) value of <35 was considered acceptable (22). More details of qRT-PCR detection (PCR amplification curves and standard curve, quality control, product sequencing verification, and stability test) are listed in Appendix S3 and Fig. S1B and C in the supplemental material.

### Modeling.

**(i) Data used for modeling.** A total of 41 EHRs, including demographic, clinical, laboratory, and radiological findings, were collected (Appendix S4), and a 20% missing value threshold was applied to remove incomplete features. Features with *P* values of <0.05 in the univariate analysis or with definite clinical significance were included for modeling. A total of 14 of the 44 original variables (41 EHRs and 3 lncRNAs) remained after filtering, including 11 EHRs and 3 lncRNAs (Appendix S4).

**(ii) Diagnostic modeling.** Multivariable logistic regression was used to develop predictive models to distinguish clinically diagnosed PTB cases from patients with suspected PTB in the selection cohort. Feature subsets were selected and compared using the best-subset selection procedure (23) and 10-fold cross-validation. The “EHR-plus-lncRNA” (EHR+lncRNA), “lncRNA-only,” and “EHR-only” models were developed according to their respective best-feature subset in the selection cohort. A cutoff for each model was determined by combining Youden’s index and a sensitivity for the samples in the training data set of ≥0.85. The models, including their cutoffs, were used for evaluation of the validation cohort.

**(iii) Nomogram presentation and evaluation.** We further adopted the nomogram to visualize the optimal model with the best AUC (24, 25). Nomogram calibration was assessed with the calibration curve and the Hosmer-Lemeshow test (a *P* value of >0.05 suggested no departure from perfect fit). The variance inflation factors (VIFs) quantified the severity of multicollinearity (a VIF of >10 indicated multicollinearity among the features in the model). Feature importance was calculated with the “varImp” function in the R package. The performance of the nomogram was tested in the validation cohort, with total points for each patient calculated. Decision curve analysis (DCA) (25) was performed by evaluating the clinical net benefit of the nomogram and EHR-only model across the overall data sets. Assessing clinical value involves comparing the nomogram and EHR-only model using the 500-bootstrap method. The nomogram was implemented as a Web-based app using R Shiny.

### Statistical analysis.

Categorical variables were analyzed by univariate analysis with a chi-square test, and continuous variables were analyzed using Mann-Whitney U tests or Student’s *t* tests. All tests were 2 sided, and *P* values of <0.05 were considered statistically significant. Modeling was constructed and validated by individuals who were blind to diagnostic categorizations.

### Data availability.

lncRNA microarray data have been deposited in the Gene Expression Omnibus under accession no. GSE119143. Sequencing data for the quantitative PCR (qPCR) products of three lncRNAs, the R code, and data for modeling are available at https://github.com/xuejiaohu123/TBdiagnosisModel.

## RESULTS

### Characteristics of prospectively enrolled participants.

The demographic and clinical characteristics of suspected clinically diagnosed PTB participants in the selection and validation cohorts are provided in Table 1. Clinically diagnosed PTB patients were younger and had higher interferon gamma release assay (IGRA) positivity rates did than their non-TB DCs (*P* value of <0.0001 for both the selection and validation cohorts), but these groups did not differ by gender, body mass index (BMI), or smoking status. Healthy subjects were age, gender, and BMI matched with PTB patients, who had significantly different blood test results than those of the PTB patients (Table 1).

**TABLE 1:** Demographic and clinical features of suspected clinically diagnosed PTB patients and healthy controls^*a*^

Clinical feature	Value for group									
	Selection cohort					Validation cohort				
	Suspected clinically diagnosed PTB patients			HSs (*n* = 578)	*P*_2_	Suspected clinically diagnosed PTB patients			HSs (*n* = 245)	*P*_4_
	Clinically diagnosed PTB cases (*n* = 141)	Non-TB DCs (*n* = 159)	*P*_1_			Clinically diagnosed PTB cases (*n* = 97)	Non-TB DCs (*n* = 140)	*P*_3_		
No. (%) of male subjects	84 (59.57)	95 (59.75)	0.976	284 (49.13)	0.026	58 (59.79)	87 (62.14)	0.715	126 (51.43)	0.162
Mean age (yr) ± SD	37.81 ± 17.93	56.68 ± 14.52	<0.0001	40.59 ± 13.11	0.084	38.29 ± 17.57	57.96 ± 16.66	<0.0001	36.82 ± 9.28	0.436
Mean BMI (kg/m^2^) ± SD	20.81 ± 2.99	20.43 ± 4.03	0.359	20.65 ± 3.19	0.57	21.59 ± 3.43	21.29 ± 3.62	0.52	21.51 ± 3.52	0.843
No (%) of smoking subjects	61 (43.26)	72 (45.28)	0.725	161 (27.85)	<0.0001	41 (42.27)	66 (47.14)	0.458	84 (34.29)	0.167
No (%) of subjects with radiological pathology	116 (82.26)	140 (88.05)	0.158			86 (88.66)	130 (92.86)	0.264		
Laboratory tests										
No. (%) of subjects with positive TB-IGRA	97 (68.88)	56 (35.22)	<0.0001			64 (66.00)	42 (30.00)	<0.0001		
Median C-reactive protein concn (mg/liter) (IQR)	16.30 (5.32–54.05)	17.80 (6.47–60.20)	0.427			13.60 (3.34–43.55)	18.60 (6.56–70.63)	0.037		
Mean hematocrit ± SD	0.37 ± 0.06	0.36 ± 0.07	0.162	0.44 ± 0.04	<0.0001	0.38 ± 0.07	0.35 ± 0.07	0.002	0.43 ± 0.04	<0.0001
Mean no. of erythrocytes (10^12^/liter) ± SD	4.33 ± 0.72	4.03 ± 0.81	0.001	4.78 ± 0.46	<0.0001	4.46 ± 0.80	3.89 ± 0.91	<0.0001	4.80 ± 0.46	<0.0001
Mean hemoglobin concn (g/liter) ± SD	122.57 ± 23.22	115.82 ± 25.20	0.017	144.46 ± 13.88	<0.0001	125.08 ± 24.25	113.11 ± 25.52	<0.0001	145.82 ± 13.73	<0.0001
Median no. of platelets (10^9^/liter) (IQR)	238.00 (177.00–305.00)	233.00 (149.00–299.00)	0.171	193.00 (158.00–223.00)	<0.0001	220.00 (160.50–313.00)	199.50 (137.00–290.75)	0.059	190.00 (165.00–230.00)	0.001
Median no. of leukocytes (10^9^/liter) (IQR)	6.03 (4.76–8.25)	6.36 (4.71–9.07)	0.488	5.92 (5.18–6.67)	0.184	6.96 (5.06–9.14)	5.93 (4.34–8.33)	0.009	5.70 (4.91–6.55)	0.217
Median no. of lymphocytes (10^9^/liter) (IQR)	1.15 (0.80–1.52)	1.28 (0.87–1.87)	0.056	1.86 (1.55–2.19)	<0.0001	1.29 (0.91–1.80)	1.22 (0.86–1.62)	0.343	1.85 (1.57–2.55)	<0.0001
Mean no. of neutrophils (10^9^/liter) (IQR)	4.02 (3.23–5.93)	4.08 (2.71–6.32)	0.956	3.47 (2.87–4.09)	<0.0001	4.03 (2.51–5.69)	4.85 (3.21–6.70)	0.023	3.36 (2.75–3.92)	0.006
Median no. of monocytes (10^9^/liter) (IQR)	0.47 (0.35–0.65)	0.42 (0.26–0.61)	0.015	0.36 (0.29–0.44)	<0.0001	0.43 (0.30–0.64)	0.46 (0.34–0.71)	0.211	0.31 (0.25–0.39)	<0.0001
Mean Alb concn (g/liter) ± SD	36.66 ± 6.78	36.60 ± 6.66	0.973	48.24 ± 2.67	<0.0001	37.33 ± 7.47	36.69 ± 7.22	0.509	47.06 ± 2.25	<0.0001
Mean globin concn (g/liter) ± SD	31.69 ± 7.66	30.68 ± 8.10	0.269	28.92 ± 3.29	0.041	30.41 ± 7.89	29.73 ± 7.91	0.514	27.41 ± 3.19	<0.0001

a Radiological pathology refers to abnormal chest imaging results, including at least one of the following signs: polymorphic abnormality, calcification, cavity, bronchus sign, and pleural effusion. Abbreviations: Alb, albumin; IQR, interquartile range; *P*_1_, *P* value for the comparison of clinically diagnosed PTB cases and non-TB DCs (nontuberculosis disease control patients) in the selection cohort; *P*_2_, *P* value for the comparison of clinically diagnosed PTB patients and healthy subjects (HSs) in the selection cohort; *P*_3_, *P* value for the comparison of clinically diagnosed PTB cases and non-TB DCs in the validation cohort; *P*_4_, *P* value for the comparison of clinically diagnosed PTB patients and healthy subjects in the validation cohort.

Clinically diagnosed PTB patients were responsible for 29.82% (238/798) of all PTB patients (238 clinically diagnosed PTB cases and 560 microbiologically confirmed PTB cases [see Appendix S1 in the supplemental material]). This rate is markedly lower than a nationwide estimate of 68% based on primary public health institutions (1) but represents the clinically diagnosed PTB rate in a referral hospital with experienced specialists.

### lncRNA microarray profiles and candidate selection.

In the screening step, microarray profiling identified a total of 325 lncRNAs that were differentially expressed (287 upregulated and 38 downregulated) in the clinically diagnosed PTB patients versus healthy subjects. Hierarchical clustering and a volcano plot revealed clearly distinguishable lncRNA expression profiles (Fig. S2). The top five lncRNA candidates were chosen based on a set of combined criteria, including a fold change of >2 between groups, a *P* value of <0.05, a signal intensity of >25 (26), and unreported lncRNAs in the TB literature (27). Three of these five lncRNAs were upregulated (*n335265*, *ENST00000518552*, and *TCONS_00013664*) and two were downregulated (*n333737* and *ENST00000497872*) in PTB versus control subjects (Table S3).

### Differentially expressed lncRNAs in clinically diagnosed PTB cases.

The expression levels of these five candidate lncRNAs were measured by qRT-PCR in the selection cohort, which consisted of 141 clinically diagnosed PTB cases, 159 non-TB DCs, and 578 healthy subjects. Two lncRNAs (*ENST00000518552* and *TCONS_00013664*) were excluded from further analysis due to their low expression levels (*C_q_* of >35) in this cohort. Of the three remaining lncRNAs, *ENST00000497872* and *n333737* were downregulated and *n335265* was upregulated in PTB patients versus healthy subjects (Fig. S3). Comparison of clinically diagnosed PTB cases and non-TB DC patients revealed decreased expression levels of *ENST00000497872* and *n333737* in PTB patients (Fig. S3) (age-adjusted *P* values of <0.0001 for both).

Short-term stability, an essential prerequisite of a potential lncRNA biomarker, was assessed in PBMC samples. This study found that incubation for up to 24 h had a minimal effect on the expression of *ENST00000497872*, *n333737*, and *n335265* (Table S4), in accordance with a previous report of lncRNA stability in blood (28).

### Diagnostic modeling and nomogram visualization.

**FIG 2:**
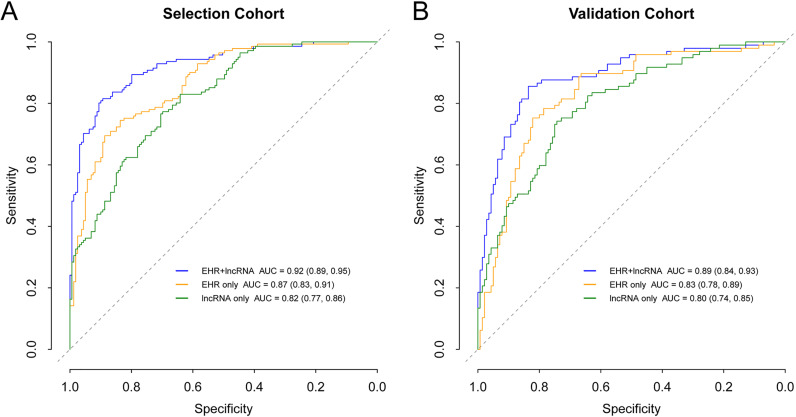
Receiver operating characteristic (ROC) curves of different models in predicting clinically diagnosed PTB from suspected patients. (A) ROC curves of the selection cohort between clinically diagnosed PTB cases and non-TB disease controls. The 10-fold cross-validation ROC curve of the EHR+lncRNA model is provided in Fig. S4 in the supplemental material. *P* values for model AUC comparisons in the selection cohort were 0.00012 (EHR+lncRNA versus EHR only), 1.402 × 10^−7^ (EHR+lncRNA versus lncRNA only), and 0.103 (EHR only versus lncRNA only). *P* values of <0.016 (0.05/3, i.e., alpha divided by the comparison number) were considered statistically significant. (B) ROC curves of the validation cohort between clinically diagnosed PTB cases and non-TB disease controls. *P* values for model AUC comparisons in the validation cohort were 0.004 (EHR+lncRNA versus EHR only), 0.0003 (EHR+lncRNA versus lncRNA only), and 0.361 (EHR only versus lncRNA only).

Fourteen features for eligible suspected patients were used for modeling, including 11 EHR features and 3 lncRNAs. Three logistic regression models, EHR+lncRNA, EHR only, and lncRNA only, were evaluated as part of the training step in the selection cohort (Appendix S4). The VIF between the features ranged from 1.02 to 1.29, indicating no collinearity within models. The EHR+lncRNA model included six EHR features and three lncRNAs. The EHR+lncRNA model yielded the highest AUC (0.92) for distinguishing clinically diagnosed PTB from suspected PTB patients, compared to AUCs of 0.87 and 0.82 for the EHR-only and lncRNA-only models, respectively (Fig. 2A). The EHR+lncRNA model also had the best performance in sensitivity, specificity, accuracy, positive predictive value, and negative predictive value (Table 2).

**TABLE 2:** Performances of the comparative diagnostic models in the selection and validation cohorts^*a*^

Model	Value (95% CI)				
	Sensitivity	Specificity	Accuracy	Positive predictive value	Negative predictive value
Selection cohort					
Clinically diagnosed PTB cases vs non-TB DCs					
EHR+lncRNA (nomogram)	0.89 (0.82–0.93)	0.80 (0.73–0.85)	0.84 (0.80–0.88)	0.80 (0.73–0.85)	0.89 (0.83–0.93)
EHR only	0.89 (0.83–0.93)	0.62 (0.54–0.68)	0.75 (0.69–0.79)	0.67 (0.60–0.74)	0.87 (0.79–0.91)
lncRNA only	0.85 (0.76–0.88)	0.55 (0.46–0.61)	0.69 (0.63–0.74)	0.62 (0.55–0.69)	0.80 (0.72–0.86)

Validation cohort					
Clinically diagnosed PTB cases vs non-TB DCs					
EHR+lncRNA (nomogram)	0.86 (0.77–0.90)	0.82 (0.75–0.87)	0.84 (0.78–0.88)	0.77 (0.68–0.83)	0.89 (083–0.93)
EHR only	0.89 (0.82–0.94)	0.65 (0.56–0.72)	0.75 (0.69–0.81)	0.64 (0.56–0.72)	0.90 (0.83–0.94)
lncRNA only	0.85 (0.76–0.90)	0.54 (0.47–0.62)	0.67 (0.60–0.73)	0.56 (0.48–0.63)	0.83 (0.75–0.89)
Microbiologically confirmed PTB cases vs non-TB DCs					
EHR+lncRNA (nomogram)	0.85 (0.81–0.88)	0.81 (0.76–0.85)	0.83 (0.80–0.86)	0.85 (0.81–0.89)	0.80 (0.75–0.84)
EHR only	0.86 (0.82–0.89)	0.63 (0.58–0.68)	0.76 (0.73–0.79)	0.75 (0.71–0.79)	0.77 (0.72–0.82)
lncRNA only	0.86 (0.82–0.89)	0.55 (0.50–0.61)	0.73 (0.69–0.76)	0.71 (0.67–0.75)	0.75 (0.69–0.81)

a Note that the cutoff probabilities in the selection cohort were 0.37 for the EHR+lncRNA model, 0.26 for the EHR-only model, and 0.32 for the lncRNA-only model. Features in each model are provided in Appendix S4 in the supplemental material. The EHR+lncRNA formula that was developed to classify patients as PTB cases or non-TB disease controls was −3.32 − 0.053 × (age) − 0.94 × log(*ENST00000497872*) − 0.39 × log(*n333737*) + 1.51 × (CT calcification) + 1.16 × (TB-IGRA) + 1.09 × (low-grade fever) + 0.014 × (hemoglobin) + 0.23 × log(*n335265*) + 0.43 × (weight loss).

**FIG 3:**
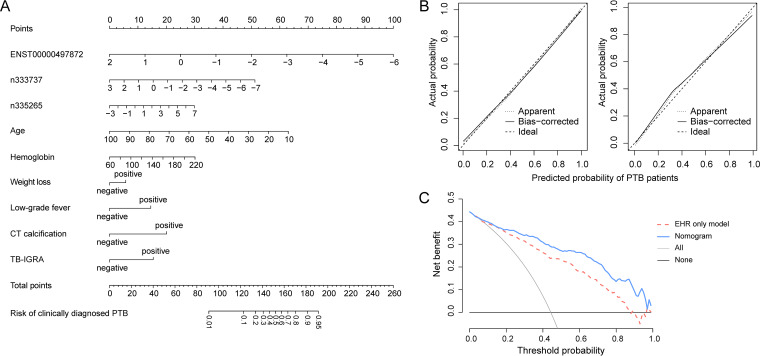
Nomogram for the prediction of clinically diagnosed PTB patients. (A) Nomogram to predict the risk of clinically diagnosed PTB patients, in which points were assigned based on the feature rank order of the effect estimates. A vertical line is drawn between the “Points” axis and the corresponding point for each feature to generate a total point score and PTB probability. (B) Calibration plot in the selection cohort (left) and validation cohort (right), with lines indicating the ideal, apparent, and bias-corrected predictions of the nomogram. (C) Decision curve analysis for the nomogram and EHR-only model, with lines indicating the nomogram, the EHR-only model, and assumptions that no patients or all patients have PTB.

The optimal EHR+lncRNA model with nine features was displayed as a nomogram (Fig. 3A), and the top five features of the nomogram were *ENST00000497872*, age, *n333737*, calcification detected by computed tomography (CT calcification), and TB-IGRA results (Table S5). The sensitivity and specificity of the nomogram for the prediction of clinically diagnosed PTB were 0.89 (95% CI, 0.82 to 0.93) and 0.80 (95% CI, 0.73 to 0.85), respectively, at a cutoff of 0.37 (Table 2). A calibration curve in the selection cohort (Fig. 3B) indicated good agreement between the nomogram prediction and actual PTB cases, which was confirmed by a nonsignificant Hosmer-Lemeshow test (*P* value of 0.957). This nine-feature nomogram was generated as a free online app (available at https://xuejiao.shinyapps.io/shiny/) to facilitate its access for other studies. This app allows the user to insert the values of specific predictors and provides the risk prediction as a whole-number percentage.

### Validation for lncRNAs and the nomogram.

In the validation step, the three candidate lncRNAs were analyzed in 97 clinically diagnosed PTB cases, 140 non-TB DCs, and 245 healthy subjects. This analysis showed an lncRNA expression pattern similar to that observed in the selection cohort (Fig. S3). All three models were applied to clinically diagnosed PTB patients and non-TB DCs of the validation cohort, and as reported in Table 2 and Fig. 2, it was found that the nomogram achieved superior discrimination (AUC, 0.89 [range, 0.84 to 0.93]) and good calibration (Fig. 3B) (*P* value of 0.668 by the Hosmer-Lemeshow test) for clinically diagnosed PTB prediction. The sensitivity and specificity of the nomogram at a cutoff of 0.37 in the validation cohort were 0.86 (range, 0.77 to 0.90) and 0.82 (range, 0.75 to 0.87), respectively. DCA indicated that the nomogram outperformed the conventional EHR-only model, with a higher clinical net benefit within a threshold probability range from 0.2 to 1 (Fig. 3C).

We also validated the nomogram in microbiologically confirmed PTB and smear-negative PTB patients. A total of 392 microbiologically confirmed PTB patients were enrolled in the validation cohort, and 48.47% of these confirmed PTB patients were smear-negative PTB cases (*n* =190). The nomogram had good discriminative power for both microbiologically confirmed PTB (AUC of 0.90) and smear-negative PTB (AUC of 0.91) patients, similar to that observed for the prediction of clinically diagnosed PTB patients (Table 2, Fig. S5 and S6, and Table S6).

### lncRNA response to anti-TB treatment.

**FIG 4:**
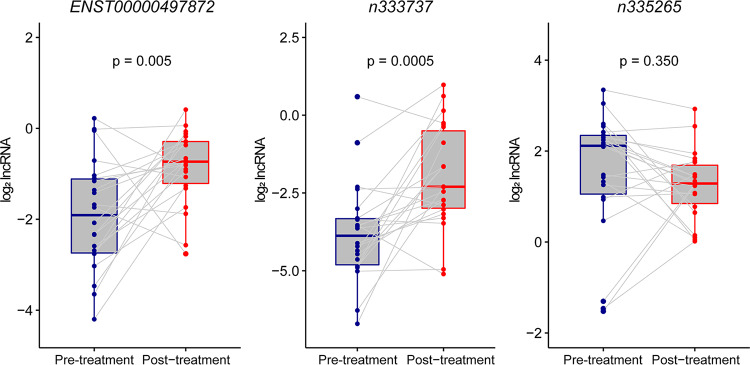
Alteration of lncRNAs before and after 2 months of intensive therapy. Shown are lncRNA expression levels before (blue) and after (red) a 2-month intensive anti-TB treatment regimen. Altered lncRNA expression levels were calculated using log_2_ lncRNA (posttreatment expression/pretreatment expression) values, and the Wilcoxon matched-paired rank test was used for comparisons among 22 paired samples. The median (interquartile range) log_2_ lncRNA values are as follows: −1.91 (−2.74, −1.11) before and −1.55 (−2.61, −0.79) after treatment for *ENST00000497872*, −3.88 (−4.81, −3.33) before and −2.30 (−2.99, −0.50) after treatment for *n333737*, and 2.12 (1.05, 2.34) before and 1.29 (0.85, 1.69) after treatment for *n335265*.

lncRNAs were next analyzed for the ability to predict anti-TB treatment response. Paired samples were collected from 22 clinically diagnosed PTB patients before and after 2 months of intensive therapy (29), and the expression levels of *ENST00000497872*, *n333737*, and *n335265* were measured by qRT-PCR. All these patients had a good response to therapy based on the clinical and radiological findings, and *ENST00000497872* and *n333737* levels were significantly increased posttreatment (*P* values of 0.005 and 0.0005, respectively) (Fig. 4), suggesting that lncRNA expression increased in response to therapy.

## DISCUSSION

The present work focused on the challenge of accurately diagnosing PTB patients without microbiological evidence of M. tuberculosis infection. To our knowledge, little literature has interrogated the exact epidemiology and diagnostic models for this subtype of PTB. We first developed and validated a novel nomogram incorporating lncRNA signatures and conventional EHR features, which can effectively discriminate clinically diagnosed PTB patients from patients with suspected diseases.

We found that three lncRNAs (*ENST00000497872*, *n333737*, and *n335265*) were potential biomarkers for clinically diagnosed PTB patients. The addition of three lncRNAs (*ENST00000497872*, *n333737*, and *n335265*) to a conventional EHR model improved its ability to identify PTB cases from suspected TB cases, with the AUCs increasing from 0.83 to 0.89. Two lncRNAs were close to immune-related genes: *ENST00000497872* (chromosome 14 [chr14], positions 105703964 to 105704602) is located close to *IGHA1* (chr14, positions 105703995 to 105708665), and *n333737* (chr14, positions 21712368 to 21712835) overlaps the *TRAV12-2* gene (chr14, positions 21712321 to 21712843). Consistent with previously reported lncRNA data (8–12, 30), these data provide new clues that lncRNAs may participate in TB immunoregulation and serve as promising biomarkers for TB diagnosis.

In addition to the three lncRNAs, we identified six EHR predictors (age, CT calcification, positive TB-IGRA, low-grade fever, elevated hemoglobin, and weight loss) that were essential in TB case finding, as proposed by previous studies (15, 16). Age was an important negative predictor for clinically diagnosed PTB, which appears to conflict with the consensus that advanced age correlates with higher TB susceptibility (31). This may be explained by differences in the enrollment of the PTB patients and control subjects. Previous studies included healthy and/or vulnerable subjects as controls, while we enrolled inpatients with a wide range of pulmonary diseases and of older ages as disease controls.

This study serves as a first proof-of-concept study to show that integrating lncRNA signatures and EHR data could be a more promising diagnostic approach for PTB patients with negative microbiological evidence of TB. The EHR+lncRNA model had good discrimination (through AUC and diagnostic parameters), reliable calibration (via a calibration curve and a Hosmer-Lemeshow test), and potential clinical utility for decision-making (using DCA). Compared with the EHR-only model, the EHR+lncRNA model shows a similar sensitivity and a significantly higher specificity in both clinically diagnosed PTB and microbiologically confirmed PTB patients, which may perform better as a “rule-in” test (32) and offer clinician confidence in a TB diagnosis and anti-TB treatment plan. In addition, the EHR+lncRNA model avoided some common problems associated with sputum-based features, such as poor sputum quality or problematic sampling (33), to improve its reliability and clinical utility.

Nomograms have been shown to remarkably promote the early diagnosis of intestinal tuberculosis (24) and prognosis prediction in PTB (34) and TBM (35). The EHR+lncRNA model here was visualized as a nomogram and further implemented in an app. The online nomogram uses readily obtainable predictors and automatically outputs a personalized quantitative risk estimate for PTB. The utilization of this user-friendly tool may speed up confirmation of a TB diagnosis, especially in resource-constrained areas with a high TB prevalence.

Our study has several limitations. Modeling in this study was conducted based on data from a single large hospital, and multicenter validation studies are needed. Furthermore, this nomogram relies on tests, including lncRNA detection and TB-IGRA, that may not be available in most community hospitals; however, the TB-IGRA and the lncRNA assay are both blood tests and can therefore be sent to a centralized facility for testing, reducing the need for specialized laboratory testing in community hospitals.

In summary, a novel nomogram that we developed and validated in this study that incorporates three lncRNAs and six EHR fields may be a useful predictive tool in identifying PTB patients with negative microbiological evidence of TB and merits further investigation.

## Supplementary Material

Supplemental file 1

Supplemental file 2

Supplemental file 3
