# Supplementary material for: Long Noncoding RNA and Predictive Model To Improve Diagnosis of Clinically Diagnosed Pulmonary Tuberculosis
Source: J Clin Microbiol. 2020 Jun 24;58(7):e01973-19. doi: 10.1128/JCM.01973-19 (PMC7315016; doi:10.1128/JCM.01973-19)
Supplement: Supplemental file 1 [file JCM.01973-19-s0001.pdf]

Supplemental Methods (e-Appendix 1–4)

e-Appendix 1. Patients' recruitment, diagnostic pipeline and management, classification

e-Appendix 2. Sample size calculation for the present study

e-Appendix 3. Detailed methodology of qRT-PCR detection

e-Appendix 4. EHR and lncRNA data collection, data-preprocessing, features before and involving in models, and modeling

e-Appendix 1. Patients' recruitment, diagnostic pipeline and management, classification

1.1 Patient recruitment

A total of 2321 inpatients with clinical suspicion of PTB patients were interrogated. Inpatients had to present one or more of the following symptoms for more than one week: cough, expectoration, fever, unexplained weight loss (3 kg or more in a month), night sweats, dyspnea of any duration, and fatigue. After screening 2321 inpatients, 561 suspected clinically diagnosed PTB patients (311 in the Selection Cohort and 250 in the Validation Cohort) satisfied all the inclusion criteria and were preliminarily enrolled. Inclusion criteria for highly suspected patients comprised: (a) new patients with clinical-radiological suspicion of PTB, (b) anti-TB therapy < 7 days on admission, (c) patients without MTB microbiologic evidence (i.e., at least two consecutive negative smear, one negative MTB-DNA PCR, one culture result), (d) age ≥ 15 years, (e) patients without severe asthma, severe immunosuppressive disease, HIV infection, cardiac or renal failure.

1.2 Diagnostic pipeline and management of patients

Demographic data and clinical symptoms of patients were obtained at the time of admission. Chest CT scan was performed according to routine care and assessed by an expert panel consisting of an experienced radiologist and a TB specialist, who were blinded to the diagnoses made by the clinicians involved in the study.

For laboratory examinations, about 3-6 ml of sputum or lower respiratory tract specimens were collected from suspected patients in a standard manner and processed

for the following tests: (a) MTB tests, at least 2-3 sputum smears for Ziehl-Neelsen staining, a culture, and an MTB-DNA PCR for each patient were done to identify the existence of MTB. (b) Others: Gram stain for bacteria, fungal culture, viral PCR test. Ten to fifteen milliliters of blood were sampled from every patient for routine tests, including standard biochemical and immunologic tests, a complete blood count test, HIV and hepatitis virus serological tests, and the following tests when necessary: serum tumor marker and autoantibodies testing. Additionally, bronchial brushings and/or tissue biopsy were considered if necessary. All the laboratory tests as mentioned above were performed before starting standard treatment by the College of American Pathologists (CAP)-certified central lab at West China Hospital.

Suspected patients first received a course of broad-spectrum antibiotics (without fluoroquinolones) for 7-14 days. Patients who clinically improved, (i.e., who reported a significant reduction of constitutional and respiratory symptoms, radiological and laboratory examinations) were considered as non-TB patients and were included as disease controls after further diagnostic confirmation. Patients who remained symptomatic after a broad-spectrum antibiotic trial were further evaluated through repeated MTB tests, chest radiograph, and physician assessment. Decisions to start empirical anti-TB therapy were made by the attending physicians with a standard anti-TB regimen and adjunctive therapy according to clinical protocol and guideline [1]. Patients were evaluated every week during hospitalization and monthly thereafter through outpatient follow-up or telephone interview.

1.3 Patients' classification

A panel of experts consisting of two experienced pulmonologists reviewed all presumed PTB cases, and final diagnoses for all cases were based on the combination of clinical assessment, radiological and laboratory results, response to the treatment, and 12-month follow-up observations.

Following the Chinese diagnostic criteria for PTB (WS288–2008) [2], clinically diagnosed PTB patients were diagnosed based on the combination of the following criteria: (a) clinical symptoms suggestive of active PTB; (b) chest imaging or bronchoscope showing lesions of active PTB; (c) repeated smear microscopy, culture and NAAT negative for MTB, (d) appropriate response to anti-TB therapy, (e) exclusion of other lung diseases, (f) others: positive anti-TB immune response (using PPD test, IGRA or anti-TB antibody test), histopathological extra-pulmonary evidence for extra-PTB.

Subjects for whom (a) MTB tests were negative, (b) confirmation of an alternative diagnosis was made, and/or (c) response to therapy was documented with a lack of anti-TB treatment or follow-up observation were considered as non-TB disease control (non-TB DC) patients.

A 12-month follow-up through telephone or WeiChat was used to further confirm the classification of PTB and non-TB patients. A total of 24 patients (11 in the Selection Cohort and 13 in the Validation Cohort), who did not consent to participate, remained an uncertain diagnosis, or lost to follow-up, were further excluded.

The 300 highly suspected patients in the Selection Cohort were divided as 141 clinically diagnosed PTB patients and 159 non-TB DC patients, and the 237 highly

suspected patients in the Validation Cohort were divided as 97 clinically diagnosed PTB and 140 non-TB DC. In addition, among 2321 inpatients, a total of 798 PTB patients were finally confirmed in our study population, and clinically diagnosed PTB patients (141 in the Selection Cohort and 97 in the Validation Cohort) accounted for 29.8% (238/798) of all the PTB patients. We also enrolled microbiologically-confirmed PTB cases in the Validation Cohort to test the predictive performance of lncRNA and models.

We summarized the above information of participant inclusion and diagnostic classification of individuals as the following flow diagram.

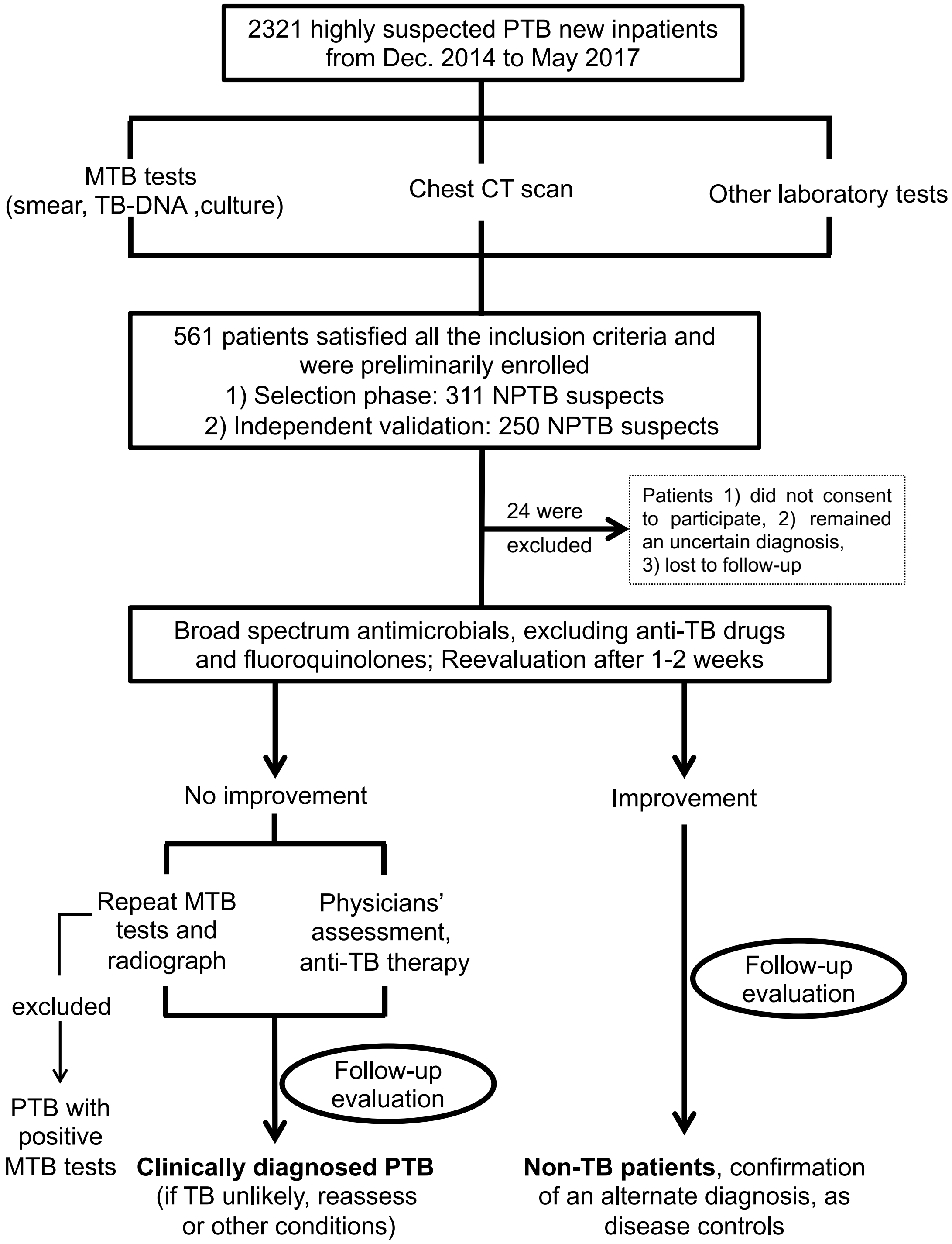

Reference

1. Jacobson KR. Tuberculosis. Annals of Internal Medicine 2017;166:ITC17-ITC32.
2. Ministry of Health, China. WS 288–2008 Diagnostic criteria for pulmonary tuberculosis Beijing, China: People's Medical Publishing House; 2008. (in Chinese)

e-Appendix 2. Sample size calculation for the present study

The sample size for the present study was performed using the HyLown Power and Sample Size Calculators (http://powerandsamplesize.com/Calculators/), which can simultaneously determine the error rate and statistical power. Setting the threshold at an error rate of 0.05 and a power of 80%, a sample size of at least 82 patients was required for lncRNA detection in the Selection Cohort and Validation Cohort, respectively. The present sample size incorporated the need for at least 10 clinically diagnosed PTB patient events per predictive variable for binary logistic regression analysis [3].

Reference

3. Griesel R, Stewart A, van der Plas H, Sikhondze W, Rangaka MX, Nicol MP, Kengne AP, Mendelson M, Maartens G. Optimizing tuberculosis diagnosis in human immunodeficiency virus–infected inpatients meeting the criteria of seriously ill in the world health organization algorithm. Clinical Infectious Diseases 2017;66:1419-1426.

e-Appendix 3. Detailed methodology of qRT-PCR detection

Candidate lncRNAs were detected using the qRT-PCR method in the Selection Cohort, and the significantly up-or down-expressed lncRNAs in the Selection Cohort were further validated in an independent cohort.

3.1 RNA isolation

Peripheral blood mononuclear cell (PBMC) samples were freshly isolated from 3 ml peripheral blood from every participant using a Human Lymphocyte Separation Tube Kit (Dakewe Biotech Company Limited, China). Total RNA was extracted from the PBMC samples using Trizol reagent (Invitrogen, USA), according to the manufacturer's instructions. The RNA pellet was dissolved in RNA Storage Solution and stored at -80 °C until use. RNA concentration and purity were evaluated by using a NanoDrop ND-1000 (Thermo Fisher Scientific, USA). Qualified RNA had an A260/A280 ratio of 1.8-2.2 and an A260/A280 ratio higher than 1.8 RNA quality was further evaluated by 1% agarose gel electrophoresis, which revealed two clear bands corresponding to 28S and 18S ribosomal RNA (rRNA). No higher molecular weight bands (above 28S rRNA band) also indicated the RNA was not contaminated with gDNA, as can be seen in Figure S1A.

3.2 Reverse transcription

The PrimeScript^{TM} RT reagent Kit with gDNA Eraser (Takara, Japan) was used to remove gDNA and synthesize cDNA. Briefly: 2 μl total RNA were added to 8 μl of the Master mix (5X gDNA Eraser buffer 2 μl, gDNA Eraser 1 μl, and RNase-free water 5 μl). After incubation at room temperature for 5 min, the 10 μl total RNA mix were added to 10 μl of the RT reaction mix (PrimeScript RT Enzyme Mix I 1 μl, RT Primer Mix 1 μl, 5X PrimeScript Buffer II 4 μl, and RNase-free water 4 μl). Reverse transcription was performed using an ABI 9700 Thermal Cycler (Applied Biosystems, USA) to produce cDNA.

3.3 qRT-PCR detection

LncRNA expressions were measured using the SYBR® Green PCR Kit (Takara, Japan) and LightCycler® 480 system (Roche, Switzerland). Specific primers are in Table S2. A final 10 µl reaction mixture for qRT-qPCR included 5 µl of SYBR® Premix Ex Taq™ II (Takara, China), 0.5 µl of 10 µM forward and reverse primer, 3 µl of ddH_2O (PCR grade), and 1 µl of template cDNA. The cycling program consisted of 95 ºC for 1 min, followed by 35 cycles at 95 ºC for 10 s, 65 - 0.5 ºC/cycle for 30 s and 72 ºC for 60 s. The samples were denatured at 95 °C for 30 s and then heated to 65 °C for 30 s at a rate of 0.2 °C/s. PCR curves are shown in Figure S1B. Fluorescence was measured to generate a high-resolution melting curve to analyze the purity of the amplification products (single peak means a high purity of PCR product), and for a more accurate PCR product validation, we further sequenced candidate lncRNAs in several random samples, which can be found at the following link https://github.com/xuejiaohu123/TBdiagnosisModel, and their peak figure in sequencing can be seen in the figure below.

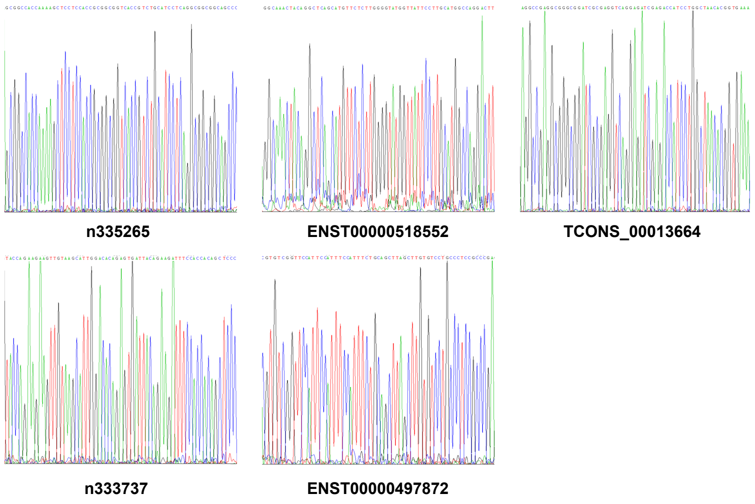

The no-cDNA template negative control, reverse transcription negative control, and blank ddH_2O control were tested, and all the above negative controls in each reaction showed no detectable signals, ensuring the lack of contamination or nonspecific products. Each sample was run in triplicate, and a CV < 15% cutoff was established to validate the technical replicates. Raw PCR data were analyzed as Cq values using Gene Scanning v1.2 software, and Cq value < 35 was considered acceptable. LncRNA expressions were measured in a blinded fashion, normalized to the endogenous control *GAPDH*, and calculated according to the $2^{-\Delta\Delta Cq}$ method, where $\Delta Cq = Cq_{\text{lncRNA}} - Cq_{GAPDH}$, $\Delta\Delta Cq = \Delta Cq - \Delta \text{average } Cq_{\text{(healthy subjects)}}$.

To estimate reaction efficiency (E) of each assay, a ten-fold serial dilution of control cDNA using synthetic *GAPDH*, covering 5 orders of magnitude, was amplified and used to generate the standard curve. As can be seen in see Figure S1C, the standard curve showed a reaction efficiency of 2.030, R^2 of 0.9973.

We also assessed the short-term stability of lncRNA expressions in PBMC. We randomly selected blood samples from 4 individuals for incubation at 4 °C for 0 h, 1 h, 2 h, 6 h, 12 h, and 24 h, respectively. These time points were chosen because blood samples were assessed within 24 h in many clinical labs in China. Table S5 shows prolonged incubation up to 24 h had minimal effect on the expression of *ENST00000497872*, *n333737*, and *n335265*.

e-Appendix 4. EHR and lncRNA data collection, data-preprocessing, features before and involving in models, and modeling

4.1 EHR and lncRNA data collection

Forty-one EHR variables for eligible suspected patients were collected before modeling, including: (a) demographic features: gender, age, BMI, and smoking; (b) clinical symptoms: cough, expectoration, chest discomfort, hemoptysis, low-grade fever, weight loss, night sweat, poor appetite, and fatigue; (c) chest imaging: polymorphic abnormality, calcification, cavity, bronchus sign, and pleural effusion; and (d) laboratory tests: TB-IGRA, hematocrit (HCT), platelets (PLT), white blood cell counts (WBC), neutrophils (N), lymphocytes (L), monocytes (M), red blood cell counts (RBC), hemoglobin (HB), albumin (ALB), globin, c-reactive protein (CRP), erythrocyte sedimentation rate (ESR), immunoglobulin A (IgA), immunoglobulin E (IgE), immunoglobulin G (IgG), immunoglobulin M (IgM), $CD3^+$ T cells, $CD4^+$ T cells, $CD8^+$ T cells, $CD4^+/CD8^+$, interleukin-6, and procalcitonin (PCT). The lncRNAs for eligible suspected patients included *ENST00000497872*, *n333737*, and *n335265*.

4.2 Data-preprocessing, and features before modeling

To make full use of data, we used the log2 values of lncRNA expressions and original EHR data in modelling. We applied a 20% missing value as thresholds to remove incomplete and uninformative EHR and lncRNA features. Univariate logistic regression and correlation analysis were conducted to select the statistical features and to avoid multicollinearity, respectively. The features with a p-value < 0.05 and/or

clinical significance were included in the subsequent model. A total of 14 out of 44 features for eligible suspected patients remained after this filtering step, including (a) 11 EHR features: age, low-grade fever, weight loss, CT calcification, CT bronchus sign, TB-IGRA, RBC, HB, WBC, L and M. (b) 3 lncRNAs: *ENST00000497872*, *n333737* and *n335265*.

4.3 Modeling

Multivariable logistic regression was used to develop predictive models to distinguish clinically diagnosed PTB from patients with suspected PTB cases in the Selection Cohort. Feature subsets were selected and compared "bestglm" R function and ten-fold cross-validation. The best feature subset of each model was determined based on the combinational criteria of discriminability (accuracy and AUC), Akaike Information Criterion and goodness of fit evaluation. The "EHR+lncRNA", "lncRNA only" and "EHR only" models were developed according to their respective best feature subset in the Selection Cohort. A cutoff of each model was determined by combining the Youden's index and the sensitivity for the samples in the training dataset equal to or greater than 0.85. The models including their cutoff were used for evaluation of the Validation Cohort.

4.4 Features included in the final models using the data from the Selection Cohort

Model	Features

EHR+lncRNA	*ENST00000497872*, *n333737*, *n335265*, age, hemoglobin, low-grade fever, weight loss, CT calcification, TB-IGRA
EHR only	age, hemoglobin, monocyte, low-grade fever, weight loss, CT calcification, CT bronchus sign, TB-IGRA
lncRNA only	*ENST00000497872*, *n333737*, *n335265*

The cutoff probability in the Selection Cohort was 0.37 for "EHR+lncRNA" model, 0.26 for "EHR only" model, and 0.32 for "lncRNA" model, respectively. The "EHR+lncRNA" formula that was developed to classify patients as PTB cases or non-TB disease controls was: -3.32 - 0.053×[age] - 0.94×log(ENST00000497872) - 0.39×log(n333737) + 1.51×[CT calcification] + 1.16×[TB-IGRA] + 1.09×[low-grade fever] + 0.014×[hemoglobin] + 0.23×log(n335265) + 0.429×[weight loss].
