# Supplementary material for: Long Noncoding RNA and Predictive Model To Improve Diagnosis of Clinically Diagnosed Pulmonary Tuberculosis
Source: J Clin Microbiol. 2020 Jun 24;58(7):e01973-19. doi: 10.1128/JCM.01973-19 (PMC7315016; doi:10.1128/JCM.01973-19)
Supplement: Supplemental file 2 [file JCM.01973-19-s0002.pdf]

Supplementary Figure 1-6

e-Figure 1. RNA electrophoresis, amplification curve of qRT-PCR and standard curve of control cDNA

e-Figure 2. Hierarchical clustering and volcano plot for differentially expressed lncRNA profiles in the Screening Cohort

e-Figure 3. LncRNA expressions between clinically diagnosed PTB patients and non-TB disease controls in the Selection and Validation Cohorts

e-Figure 4. Ten-fold cross-validation ROC of "EHR+lncRNA" model developed using the data from the Selection Cohort

e-Figure 5. The predictive performance of nomogram for microbiologically-confirmed PTB patients

e-Figure 6. The predictive performance of nomogram for smear-negative PTB patients

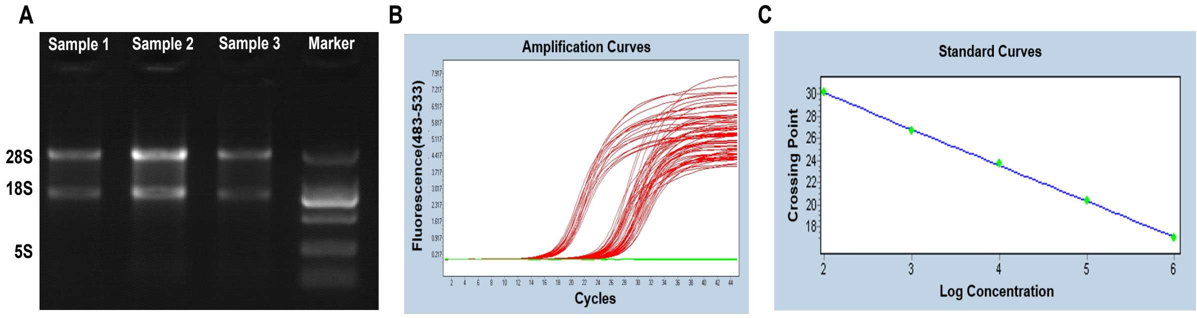

e-Figure 1. RNA electrophoresis, amplification curve of qRT-PCR and standard curve of control cDNA.

(A), RNA electrophoresis. Marker: DNA marker (100-2000bp).

(B), Amplification curve of qRT-PCR. The left set of amplification curves indicates the endogenous control *GAPDH*, and the right set of amplification curves indicates the lncRNA gene.

(C), Standard curve of control cDNA. Cq values (i.e., crossing points) are plotted versus the log10 [cDNA quantity]. Some information for the standard curve: Y = -3.252 X + 36.524, R^2 = 0.9973, reaction efficiency = 2.030, error = 0.0154.

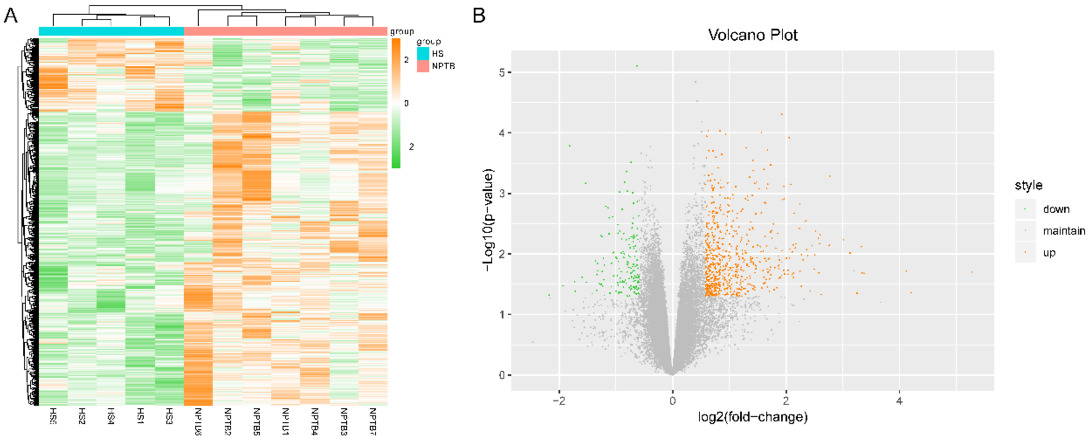

e-Figure 2. Hierarchical clustering and volcano plot for differentially expressed lncRNA profiles in the Screening Cohort.

(A), Heatmap of lncRNA profile generated by hierarchical clustering. Pink bar, clinically diagnosed PTB patients with negative MTB evidence, herein referred to as NPTB for short; cyan bar, healthy subjects (HS, for short). Downregulated lncRNAs are depicted in green and upregulated in orange.

(B), Volcano plot. The signal values were distributed in the corresponding area after the data normalization. The green dots and orange dots are significantly down- and upregulated lncRNA distributions, respectively.

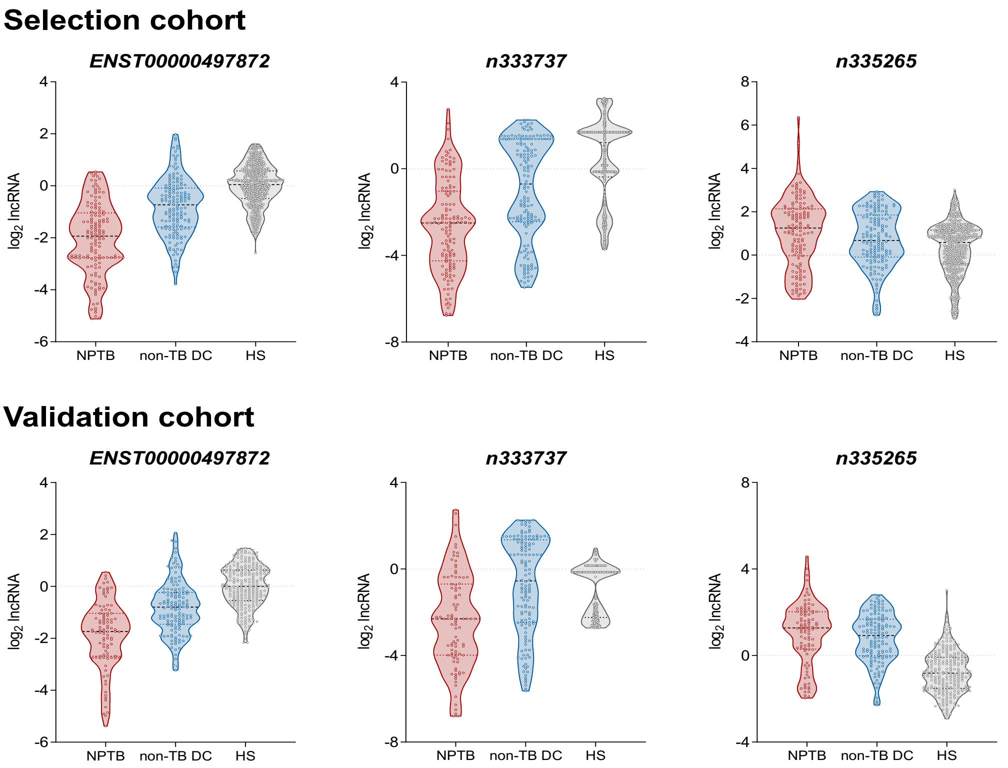

e-Figure 3. LncRNA expressions between clinically diagnosed PTB patients, non-TB disease controls and healthy controls in the Selection and Validation Cohorts. Clinically diagnosed PTB patients with negative MTB evidence, herein referred to as NPTB for short. These error bar of violin plots demonstrate the median and interquartile range. (1) Clinically diagnosed PTB *versus* non-TB disease controls (DC): *ENST00000497872* and *n333737* show statistical significance (p-value < 0.0001 after age-adjustment for both the Selection and Validation Cohorts) in group comparison, and *n335265* indicates a p-value of 0.080 and 0.110 in the Selection Cohort and Validation Cohort, respectively. (2) Clinically diagnosed PTB: *ENST00000497872* and *n333737* were downregulated and *n335265* was upregulated in clinically diagnosed PTB patients in the Selection and Validation Cohorts (p-value < 0.0001 after age-adjustment).

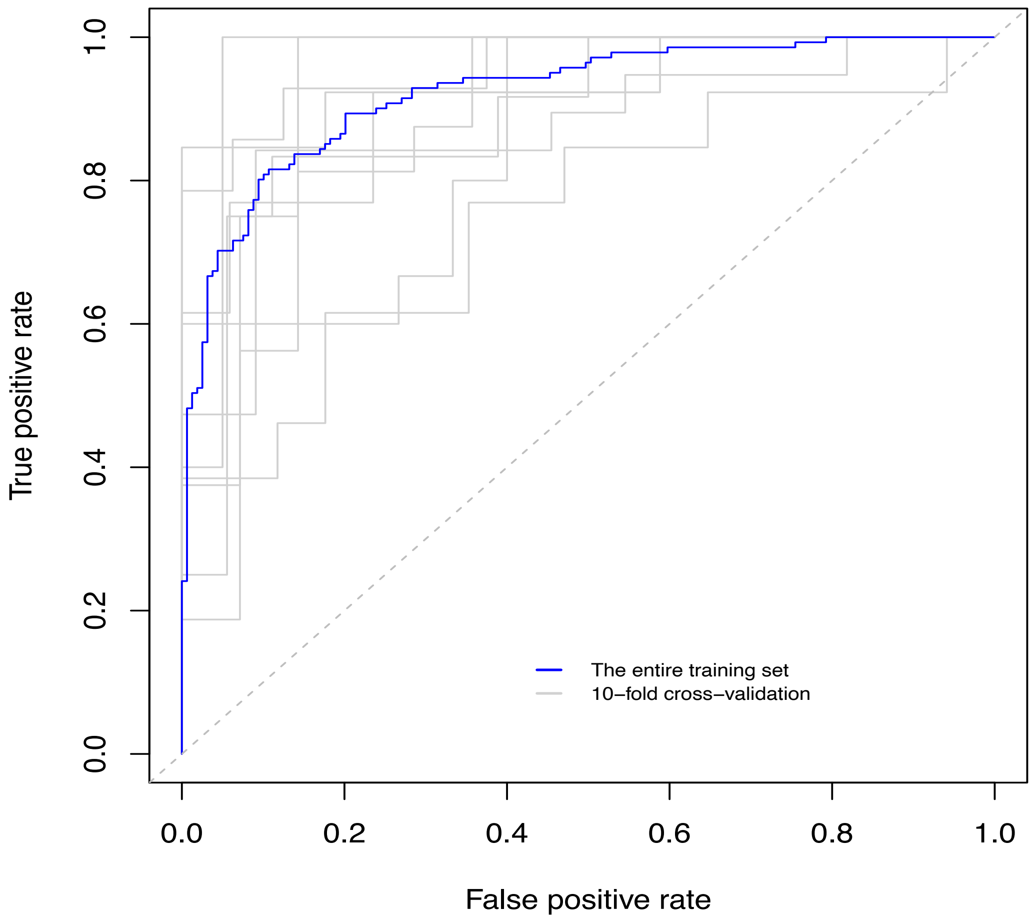

e-Figure 4. Ten-fold cross-validation ROC of "EHR+lncRNA" model developed using the data from the Selection Cohort.

The "EHR+lncRNA" models were trained using data from the Selection Cohort. The AUC of "EHR+lncRNA" models was 0.89 (0.84-0.93). Sensitivity and specificity of the "EHR+lncRNA" model for prediction of clinically diagnosed PTB was 0.89 (0.82-0.93) and 0.80 (0.73-0.85) at a cutoff of 0.37. The blue line represents the ROC for the entire training dataset. The grey lines refer to the ROC developed for each of the 10-fold cross-validations.

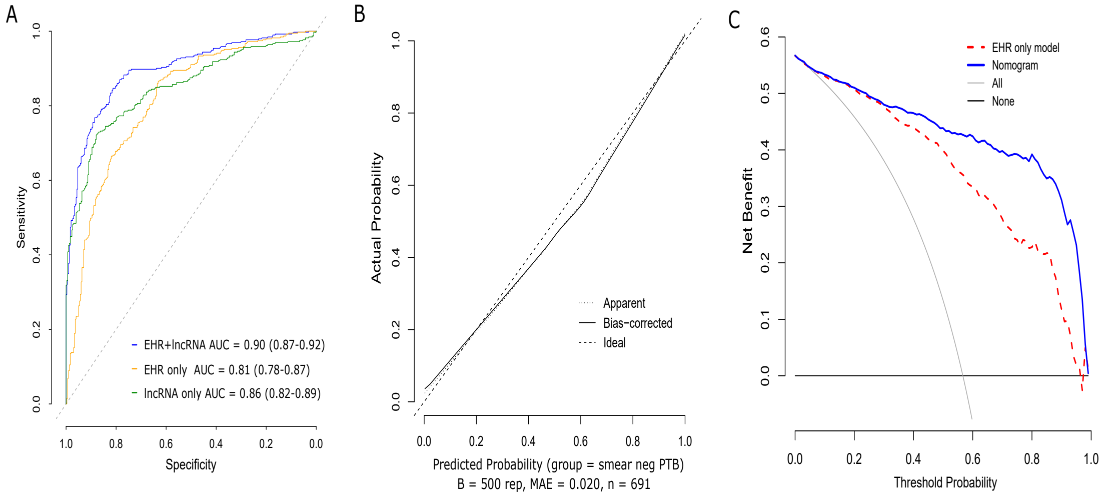

e-Figure 5. The predictive performance of nomogram for microbiologically-confirmed PTB patients

(A), AUC of different models in differentiating microbiologically-confirmed PTB patients (n = 392) from non-TB disease controls (n = 299). P-values for model AUC were: 6.771×10^{-9} ("EHR+lncRNA" vs "EHR only"), 8.437×10^{-6} ("EHR+lncRNA" vs "lncRNA only"), and 0.121 ("EHR only" vs "lncRNA only"), respectively. P-values < 0.016 (0.05/3) were considered statistically significant.

(B), Calibration plot in the microbiologically-confirmed PTB patients, with lines indicating the ideal (dashed), apparent (dotted) and bias-corrected (unbroken) predictions of the nomogram.

(C), Decision curve analysis for the nomogram and "EHR only" model in the microbiologically-confirmed PTB cases, with lines indicating the nomogram (blue), "EHR only" model (red dash), and assumptions that no patients or all patients have PTB (black and grey, respectively).

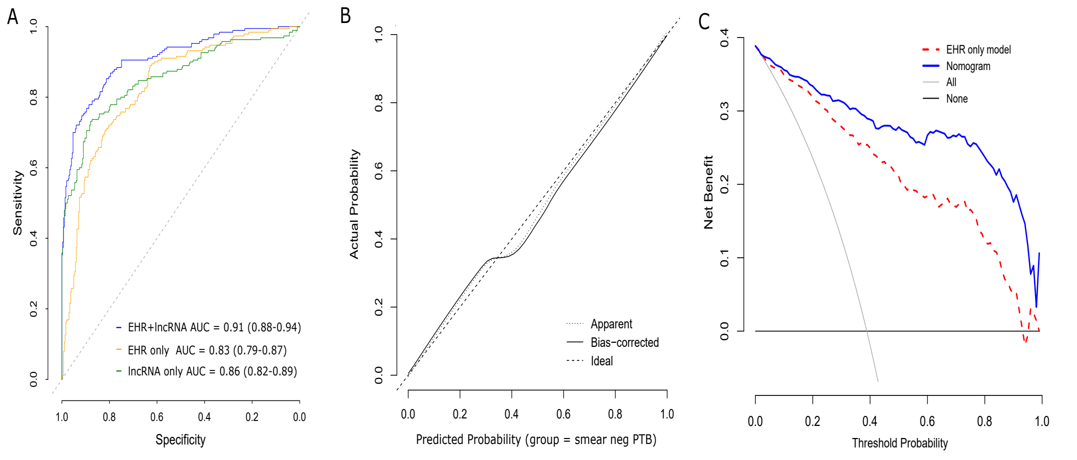

e-Figure 6. The predictive performance of nomogram for smear-negative PTB patients

(A), AUC of different models in differentiating smear-negative PTB patients (n =190) from non-TB disease controls (n = 299). P-values for model AUC were: 5.114×10^{-6} ("EHR+lncRNA" vs "EHR only"), 4.265×10^{-5} ("EHR+lncRNA" vs "lncRNA only"), and 0.336 ("EHR only" vs "lncRNA only"), respectively. P-values < 0.016 (0.05/3) were considered statistically significant.

(B), Calibration plot in the smear-negative PTB patients, with lines indicating the ideal (dashed), apparent (dotted) and bias-corrected (unbroken) predictions of the nomogram.

(C), Decision curve analysis for the nomogram and "EHR only" model in the smear-negative PTB patients, with lines indicating the nomogram (blue), "EHR only" model (red dash), and assumptions that no patients or all patients have PTB (black and grey, respectively).
