# Supplementary material for: Long Noncoding RNA and Predictive Model To Improve Diagnosis of Clinically Diagnosed Pulmonary Tuberculosis
Source: J Clin Microbiol. 2020 Jun 24;58(7):e01973-19. doi: 10.1128/JCM.01973-19 (PMC7315016; doi:10.1128/JCM.01973-19)
Supplement: Supplemental file 3 [file JCM.01973-19-s0003.pdf]

Supplementary Table 1-6

e-Table 1. Disease controls in the present study

e-Table 2. Specific qRT-PCR primers for lncRNAs

e-Table 3. The expression of five candidate lncRNAs in the Screening Cohort

e-Table 4. Short-term stability evaluation of lncRNAs in PBMC samples

e-Table 5. Details of "EHR+lnRNA" logistic regression model to differentiate clinically diagnosed PTB among 300 highly suspected patients

e-Table 6. Performances of the diagnostic models between smear-negative PTB patients and non-TB disease controls

e-Table 1. Disease control patients in the present study.

Selection Cohort<br>Non-TB disease controls (n = 159)	Validation Cohort<br>Non-TB disease controls (n = 140)
(1) Pulmonary infection (n = 155)	(1) Pulmonary infection (n = 134)
103 Pneumonia (bacterial, fungal, viral, or mixed)	84 Pneumonia (bacterial, fungal, viral, or mixed)
42 Pneumonia complicated with other conditions (sarcoidosis, COPD, bronchiectasis, interstitial lung fibrosis, or cor pulmonale)	45 Pneumonia complicated with other diseases (sarcoidosis, bronchiectasis, COPD, interstitial lung fibrosis, pneumocystis jiroveci infection, histoplasmosis, or vasculitis)
10 Others: pulmonary abscess, bronchiectasis, or pulmonary embolism, nontuberculous mycobacteria	5 Others: interstitial lung fibrosis, pulmonary abscess, bronchiectasis
(2) Lung cancer (n = 4)	(2) Lung cancer (n = 6)

e-Table 2. Specific qRT-PCR primers for lncRNAs.

Gene accession	Forward primer	Reverse primer	Amplicon length (bp)
n335265	CGCAGAAGTAAGTAGCCGGG	ACTGGATGAGCGTGAAGTGG	230
ENST00000518552	AGGCACGATTATCACTCACACACT	CCACAGAAAGATGAACCCACAGA	194
TCONS_00013664	AAGAAGGAAGACGGAGCAGC	CTCCGGTGGATCTCCGAAGT	231
n333737	GCAGAAAGCAAGGACCAA	GGATGAGCAGCGATGAAG	223
ENST00000497872	TTCCTCACCCTCTTCCTGCT	AAGGCATGTGAGTAAGGGCG	216
GAPDH	GCACCGTCAAGGCTGAGAAC	GGATCTCGCTCCTGGAAGATG	73

LncRNA primers were designed according to the lncRNA sequences from the Noncode (1) or LNCipedia database (2).

Reference

1. Fang S, Zhang L, Guo J, Niu Y, Wu Y, Li H, Zhao L, Li X, Teng X, Sun X, Sun L, Zhang MQ, Chen R, Zhao Y. Noncodev5: A comprehensive annotation database for long non-coding rnas. Nucleic Acids Research 2018; 46: D308-D314.

2. Volders P-J, Helsens K, Wang X, Menten B, Martens L, Gevaert K, Vandesompele J, Mestdagh P. Lncipedia: A database for annotated human lncrna transcript sequences and structures. Nucleic Acids Research 2013; 41: D246-D251.

e-Table 3. The expression of five candidate lncRNAs in the Screening Cohort.

LncRNA	Fold-Change	p	Gene location (hg38)
n335265	5.53	0.007	chr19:2476356-2477126
ENST00000518552	3.21	0.0002	chr8:55893595-55895739
TCONS_00013664	3.11	0.005	chr7:26392-35472
n333737	-3.30	0.028	chr14:21712368-21712835
ENST00000497872	-2.29	0.015	chr14:105703964-105704602

e-Table 4. Short-term stability evaluation of lncRNAs in PBMC samples.

LncRNA	Incubation time at 4 °C						p
	**0 h**	**1 h**	**2 h**	**6 h**	**12 h**	**24 h**	
ENST00000497872	1.65 (0.57-2.40)	1.55 (0.51-1.98)	1.31 (0.47-1.85)	1.31 (0.47-1.75)	1.19 (0.46-1.48)	1.20 (0.50-1.56)	0.088
n333737	0.26 (0.23-0.32)	0.23 (0.19-0.26)	0.21 (0.19-0.23)	0.19 (0.16-0.20)	0.18 (0.12-0.26)	0.15 (0.11-0.23)	0.075
n335265	7.10 (5.23-7.95)	6.52 (5.38-7.58)	5.54 (4.68-6.13)	5.65 (5.14-13.44)	4.96 (4.32-8.79)	5.43 (4.35-11.59)	0.471

Each sample for each lncRNA was run in triplicate. The relative expression of each lncRNA was calculated using the $2^{-\Delta\Delta Cq}$ method, and statistical analysis was performed with the Friedman test, a non-parametric alternative to the one-way ANOVA with repeated measures. A p-value < 0.05 was regarded as statistically significant.

e-Table 5. Details of "EHR+lnRNA" logistic regression model to differentiate clinically diagnosed PTB among 300 highly suspected patients.

Features	β-coefficient	Wald's p-value	Feature importance
ENST00000497872	-0.940	<0.0001	5.667
age	-0.053	<0.0001	4.846
n333737	-0.385	<0.0001	4.294
CT calcification*	1.512	<0.0001	3.966
TB-IGRA*	1.159	0.002	3.149
low-grade fever*	1.092	0.004	2.852
hemoglobin	0.014	0.063	1.861
n335265	0.225	0.087	1.712
weight loss*	0.429	0.383	0.872

*The features are two-category, and the reference is the category with negative results. Other features are continuous. Note: We evaluated the "EHR+lnRNA" model with Hosmer-Lemeshow test (p-value = 0.957), Likelihood ratio test (p-value < 0.0001), McFadden R^2 (0.491), and Nagelkerke R^2 (0.658), and these evaluations indicated no evidence of poor fit for the "EHR+lnRNA" model built in the Selection Cohort. Feature importance was calculated with the "varImp" function in R package.

e-Table 6. Performances of the diagnostic models between smear-negative PTB patients and non-TB disease controls.

Model performance	Smear-negative PTB patients *vs* non-TB disease controls		
	EHR+lncRNA (Nomogram)	EHR only	lncRNA only
Sensitivity	0.85 (0.80-0.90)	0.87 (0.82-0.91)	0.87 (0.82-0.91)
Specificity	0.81 (0.76-0.85)	0.63 (0.58-0.68)	0.55 (0.50-0.61)
Accuracy	0.83 (0.79-0.86)	0.72 (0.68-0.77)	0.68 (0.63-0.72)
Positive predictive value	0.74 (0.68-0.79)	0.60 (0.54-0.66)	0.55 (0.50-0.61)
Negative predictive value	0.90 (0.85-0.93)	0.89 (0.84-0.92)	0.87 (0.82-0.91)
